# Intergenerational Transmission of Education and ADHD: Effects of Parental Genotypes

**DOI:** 10.1007/s10519-020-09992-w

**Published:** 2020-02-06

**Authors:** Eveline L. de Zeeuw, Jouke-Jan Hottenga, Klaasjan G. Ouwens, Conor V. Dolan, Erik A. Ehli, Gareth E. Davies, Dorret I. Boomsma, Elsje van Bergen

**Affiliations:** 1 Department of Biological Psychology, Vrije Universiteit, Van der Boechorststraat 7-9, 1081 BT Amsterdam, The Netherlands; 2 Amsterdam Public Health Research Institute, VUmc, Amsterdam, The Netherlands; 3 Avera Institute for Human Genetics, Avera McKennan Hospital & University Health Center, Sioux Falls, SD USA

**Keywords:** Intergenerational transmission, Genetic nurturing, Polygenic scores, Educational attainment, Academic achievement, ADHD

## Abstract

It remains a challenge to determine whether children resemble their parents due to nature, nurture, or a mixture of both. Here we used a design that exploits the distinction between transmitted and non-transmitted alleles in genetic transmission from parent to offspring. Two separate polygenic scores (PGS) were calculated on the basis of the transmitted and non-transmitted alleles. The effect of the non-transmitted PGS is necessarily mediated by parental phenotypes, insofar as they contribute to the rearing environment of the offspring (genetic nurturing). We calculated transmitted and non-transmitted PGSs associated with adult educational attainment (EA) and PGSs associated with childhood ADHD in a general population sample of trios, i.e. child or adult offspring and their parents (N = 1120–2518). We tested if the EA and ADHD (non-)transmitted PGSs were associated with childhood academic achievement and ADHD in offspring. Based on the earlier findings for shared environment, we hypothesized to find genetic nurturing for academic achievement, but not for ADHD. In adults, both transmitted (R^2^ = 7.6%) and non-transmitted (R^2^ = 1.7%) EA PGSs were associated with offspring EA, evidencing genetic nurturing. In children around age 12, academic achievement was associated with the transmitted EA PGSs (R^2^ = 5.7%), but we found no support for genetic nurturing (R^2^ ~ 0.1%). The ADHD PGSs were not significantly associated with academic achievement (R^2^ ~ 0.6%). ADHD symptoms in children were only associated with transmitted EA PGSs and ADHD PGSs (R^2^ = 1–2%). Based on these results, we conclude that the associations between parent characteristics and offspring outcomes in childhood are mainly to be attributable to the effects of genes that are shared by parents and children.

## Introduction

Research on the influences of the family environment on children’s behavior is complicated by the fact that parents provide their offspring with both the family environment and genes, leading to intertwined effects and possible gene–environment correlation (Scarr and McCartney 1983; Plomin et al. 1977). Scarr and McCartney distinguished three types of gene–environment correlations. Passive gene–environment correlation arises when the rearing environment that parents created is related to the parents’ and hence to the children’s genotypes. Evocative gene–environment correlation arises when children’s heritable behavior evokes responses from others in their environment. Active gene–environment correlation arises when children actively seek out environments that fit their genotypes. Passive gene–environment correlation may contribute to the well-established associations between home characteristics and childhood outcomes. Examples include the association between the number of books in the home and children’s reading skills (van Bergen et al. 2017; Sikora et al. 2019) and the association between household chaos and children’s problem behavior (Coldwell et al. 2006). Specifically, an association between number of books in the home and children’s reading skills may arise, if parents, who are good and avid readers, create a book-rich home environment (Mol and Bus 2011). The biological children of these parents are the recipients of both genes and rearing environments that are beneficial for reading (van Bergen et al. 2017). Given that reading ability and, presumably, love of reading are heritable phenotypes, the book-rich rearing environment is dependent in part on the parental genotype, as is the offspring reading ability. Indeed, twin and family studies have shown that the rearing environment is subject to genetic influence (Plomin and Bergeman 1991; Vinkhuyzen et al. 2010).

Ignoring genetic influences in studying the rearing environment can lead to erroneous inference concerning the role of the environment (Hart et al. 2019). A genetic design allows us to control for children’s genetic propensities to demonstrate genuine family environmental influences (Bates et al. 2018; Kong et al. 2018). This design exploits measures genotypes of children and their parents to demonstrate the influence of the parental genotype on the children’s rearing environment. At each autosomal locus, parents transmit one of the two homologous alleles to their offspring. In genotyped parents and offspring trios it can be determined which alleles were transmitted. In the offspring, one can calculate two polygenic scores (PGS): one based on the transmitted alleles and one based on non-transmitted alleles. The PGS for educational attainment (EA) has been shown to explain ~ 12% of variance in people’s EA (Lee et al. 2018). Consistent with this, Bates et al. and Kong et al. demonstrated that the EA PGS of the transmitted alleles explained variance in offspring EA in early adulthood. But strikingly, the EA PGS based on the non*-*transmitted alleles also explained variance in offspring EA. This is interpreted to mean that the parental contribution to the offspring environment (relevant to EA) is in part a function of the parental genotype. Hence, Kong et al. termed this phenomenon ‘genetic nurturing’, which will induce a (passive) gene–environment correlation. The transmitted alleles, present in both the parents and the offspring, have a direct genetic effect on offspring’s behavior, and an indirect genetic effect that is mediated by the environment that parents create depending on their EA. Therefore, transmitted alleles have both genetic nurturing effects and direct genetic effects, while non-transmitted alleles only have genetic nurturing effects.

In the current study, we applied this design to seek proof for influences of the family environment related to parental EA and ADHD on two important and heritable childhood traits, academic achievement and ADHD. Previous research has established that ADHD and academic achievement have a strong negative link (Polderman et al. 2010). This relationship is at least partly genetic in nature, as shown in cross-domain PGS predictions: the transmitted PGS of EA predicted children’s ADHD symptoms (de Zeeuw et al. 2014) and the transmitted PGS of ADHD predicted children’s educational outcomes (Stergiakouli et al. 2017). The question remains what the role is of the home environment in this relationship.

In a classical twin design, in which it is assumed that genetic and environmental effects are uncorrelated, the common environmental factor would presumably include the effects of genetic nurturing. First, genetic nurturing is an effect of the home environment that is shared between twins. Second, genetic nurturing causes a (passive) gene–environment correlation, and if this correlation is not modeled it contributes to common environmental variance (Purcell 2002). Genetic nurturing, if present, should also be reflected by the presence of environmental transmission in extended family studies, such as adoption, parent–offspring and children-of-twins designs, as these designs can decompose parent–offspring resemblance into a part due to the family environment and a part due to genetic transmission (D’Onofrio et al. 2003). In studies utilizing a PGS from either parent, an association between maternal or paternal PGS and offspring phenotype (taking into account the effect of a child’s own PGS) would also point to genetic nurturing (Belsky et al. 2018).

With respect to academic achievement and ADHD, we entertained opposing hypotheses regarding genetic nurturing of EA and ADHD. In the Netherlands, academic achievement in primary school is substantially heritable (~ 75%) with modest common environmental influences (~ 10%) (de Zeeuw et al. 2016). Associations between parental EA and academic achievement in adopted children have been found in some, but not all, adoption studies (Lundborg et al. 2018). In addition, a parent–offspring study of EA in adulthood found both genetic and environmental transmission (McGue et al. 2017). Children-of-twins studies also indicate that intergenerational transmission of education is partly due to the effects of parental EA on the home environment (e.g. Chevalier et al. 2013). Finally, maternal PGSs for EA were significantly associated with children’s performance at the end of secondary school, taking into account the effect of the offspring PGS. The maternal PGS effect was therefore mediated by parenting behaviors (Wertz 2019). Based on these findings we expected a small role of genetic nurturing in academic achievement in children.

Heritability of ADHD is also high (~ 75%), as demonstrated in both case–control and general population samples (Faraone and Larsson 2019). Common environmental effects on ADHD, however, are found to be absent. There is some evidence that dominance does contribute to ADHD variance (Rietveld et al. 2003; Nikolas and Burt 2010). Extended family studies, which can accommodate both dominant genetic and common environmental effects, focusing on the transmission of ADHD within families are scarce (McAdams et al. 2014). A study including adult twins and their siblings as offspring established that intergenerational transmission was explained by genetic inheritance, without support for environmental transmission (Boomsma et al. 2010). A children-of-twins study (Silberg et al. 2012), also showed that the association between antisocial behavior in parents and ADHD in offspring is due solely to genetic transmission (Silberg et al. 2012). Based on these studies, we did not expect to find an effect of genetic nurturing on offspring ADHD symptoms.

The aim of the present study was to replicate the finding of an effect of genetic nurturing of EA on adult offspring EA from Kong et al. (2018) and Bates et al. (2018) in a Dutch sample. In addition, we investigated the presence of genetic nurturing of EA and ADHD on childhood academic achievement and ADHD symptoms. We considered two continuous measures of ADHD symptoms based on maternal and teacher ratings, as rater agreement between mother and teacher is relatively low (*r* = 0.44) (Achenbach & Rescorla, 2001), possibly suggesting that children’s behavior depends in part on the context (i.e., home versus school). We examined the effect of EA and ADHD PGSs, both within and between domains, based on transmitted and non-transmitted parental alleles, on offspring academic achievement and ADHD symptoms at the end of primary school. In sum, we investigated whether the transmitted PGS and non-transmitted PGS of (1) EA was associated with adults EA, (2) EA was associated with children’s academic achievement, (3) ADHD was associated with children’s ADHD symptoms, (4) EA was associated with children’s ADHD symptoms and (5) ADHD was associated with children’s academic achievement.

## Methods

### Participants

The Netherlands Twin Register (NTR) was established around 1987 by the Department of Biological Psychology at the Vrije Universiteit Amsterdam and recruits approximately 40% of new-born twins or higher-order multiples in the Netherlands for longitudinal research. Parents of twins receive a survey about the development of their children every 2 to 3 years until the twins are 12 years old. Starting at age 7, parents are asked consent to also approach the primary school teacher(s) of their twin and other children. The survey sent to mothers, fathers and teachers includes the age and context appropriate version of the Achenbach System of Empirically Based Assessment (ASEBA) (Achenbach et al. 2017). Adult twins were registered with the NTR through several approaches, including, for example, recruitment through city council offices in the Netherlands, advertising in NTR newsletters and the internet. Parents, siblings, spouses and offspring of adult twins are also invited to take part. Since 1991, participants receive a survey every 2 to 3 years with questions on, amongst others, health, personality, and lifestyle. The NTR has also been collecting genotype data in both children and adults in several large projects. More details concerning the NTR’s data collection, the methods of recruitment, participants’ background and response rates are described elsewhere (Ligthart et al. 2019).

For 5900 offspring (from 2649 families) their own, as well as the genotype data of both of their parents were available. Genotyping in the NTR has been carried out in subsamples that are, in general, unselected for phenotype. Data were excluded if an individual had a non-European ancestry (n = 472). In this group of families, information on EA was available in people over 25 years of age for 1931 adult offspring (662 males and 1260 females) from birth cohorts 1946–1991. Childhood academic achievement was assessed around age 12 and available for 1120 offspring (509 boys and 611 girls) from birth cohorts 1983–2002. ADHD symptoms were assessed at age 10 and 12. If available, age 12 data were analyzed, otherwise age 10 data. Data on ADHD symptoms at home were available for 2518 children (1202 boys and 1316 girls) from birth cohorts 1986–2008. Data on ADHD symptoms at school were available for 1969 (968 boys and 1001 girls) children from birth cohorts 1986–2011.

### Measures

#### Educational attainment

EA in adults was measured by means of a self-report on highest obtained degree. The responses were recoded into four categories: primary education (level 0), lower secondary education (level 1), higher secondary education (level 2) and tertiary education (level 3).

#### Academic achievement

Academic achievement in children was assessed by a nationwide standardized educational achievement test (Cito 2002). The results on this test are, in combination with teacher advice, used to determine the most suitable level of secondary education. Around 75% of Dutch children took this test in their final year of primary school as administration of the test was not compulsory. The test consisted of multiple choice items in four domains, namely Arithmetic, Language, Study Skills and Science and Social Studies. The first three test scales were combined into a Total Score, which was converted into a score ranging from 500 to 550, which reflects the child's standing relative to the total group of children who took the test in a given school year (van Boxtel et al. 2010).

#### ADHD symptoms

ADHD symptoms were assessed with the ASEBA system empirically based syndrome Attention Problems scale (Achenbach et al. 2017). The Child Behavior Check List (CBCL) for school aged children (6–18 years) was used to assess behavior at home, and the Teacher Report Form (TRF) for behavior at school. The ASEBA Attention Problems scale includes items (CBCL: 10 items and TRF: 26 items) on inattention (e.g. ‘Fails to finish things he/she starts’) and hyperactivity/impulsivity (e.g. ‘Can’t sit still’). The items are scored on a 3 point scale from 0 (‘not true or never’) to 2 (‘completely true or very often’). Missing items were imputed by the average item score of the scale for a child if missingness on the scale items was less than 20%. The data showed an L-shaped distribution and were square root transformed prior to analyses.

#### Genotyping

The genotype data used for this study included 17,620 unique DNA samples, done on several different platforms: Affymetrix–Perlegen (n = 1117), Illumina 660 (n = 1323), Illumina Omni Express 1 M (n = 234), Affymetrix 6.0 (n = 7086), Affymetrix Axiom (n = 2665) and Illumina GSA (n = 5195). Genotype calls were made with the platform specific software (Birdseed, APT-Genotyper, Beadstudio) following manufacturers' protocols. For the Affymetrix-Perlegen and Illumina 660 platforms, the single nucleotide polymorphisms (SNPs) were lifted over to build 37 (HG19) of the Human reference genome.

Per platform, a sample was removed if the call rate for this person was < 90%, the Plink 1.07 F heterozygosity value was < − 0.10 or > 0.10, the gender of the person did not match the DNA of the person, the IBD status did not match the expected familial relations, or the sample had > 20 Mendelian errors. In case a subject was genotyped on multiple platforms, only the platform with the highest number of SNPs was selected if genotypes were concordant (> 0.97). Allele and strand alignment of SNPs was done against the Dutch Genome of the Netherlands (GONL) reference panel for each platform (Boomsma et al. 2014). SNPs were removed in each platform when Minor Allele Frequency (MAF) < 0.01, Hardy–Weinberg Equilibrium (HWE) test p-value < 10^–5^ or the call rate of the SNP was < 95%. Subsequently, only SNPs were selected if the allele frequency of the SNP deviated < 0.10 as compared to the GONL data. All palindromic SNPs with a MAF > 0.40 were also removed. The individual platform data were then merged into a single dataset. In this dataset, the sample IBD was re-compared with their expected familial relations and samples were removed if these did not match. Because the number of completely overlapping SNPs within this combined set off platforms is too small (~ 70 K) for imputation against 1000G, the data were first phased and imputed with Mach-admix, using GONL as a reference panel. This was done for those SNPs that survived quality control and were present on at least one platform, forcing missing genotype imputation for all SNPs. Best guess genotypes were generated from these data, and the following SNPs were selected: SNPs with a R^2^ > 0.90, with HWE p > 10^–5^, with a Mendelian error rate < 2%, and if the association of one platform = case vs. the other platforms = controls p-value > 10^–5^ (applied for each platform) resulting in a genetic backbone of 1.2 M SNPs. After this step, 3017 DNA confirmed monozygotic twin samples were returned into the dataset by duplicating the SNP data of their co-twin. Another 364 DNA samples were added, 335 out of the original 349 samples, plus 29 of their confirmed monozygotic twins, of the NTR that were also sequenced in the GONL reference population. The resulting was a final dataset of 21,001 individuals from 6671 families with 1.2 M SNPs. The cross-chip imputed data were used to calculate genetic principal components using SmartPCA software, and the PCAs were subsequently used to determine if a person was from non-European descent (Galinsky et al. 2016)*.* The full set with 1.2 M SNPs was then aligned against the 1000 genomes phase 3 version 5 reference panel, and imputed on the Michigan imputation server (Das et al. 2016). From the imputed 1000G VCF files, best guess genotypes were calculated for all markers using Plink 1.96.

#### Non-transmitted genotypes

In total there were 2649 families having two genotyped parents, with 5900 offspring, including 1245 MZ twin pairs, for which allele transmission could be calculated on the 1000G imputed data. Before this calculation, the genotype data were filtered using the following criteria: only ACGT SNPs on the autosomes, no SNPs with duplicate positions, no SNPs with 3 or more alleles, MAF > 0.01, HWE p > 10^–5^ and genotype call rate > 0.99, leaving 7,411,699 SNPs. For the 5900 offspring, this is the transmitted alleles dataset. Subsequently, all children were defined as being a case, and then the Plink–tucc option was used to generate a single TDT pseudo-control genotype for each child (given the 2 parents), resulting in the non-transmitted alleles dataset. Both datasets were then used to calculate PGSs.

#### Polygenic scores

For the EA PGS calculation we used the GWA summary statistics from the EA meta-analysis (Lee et al. 2018) and for ADHD we used the statistics from the meta-analysis for ADHD (Demontis et al. 2019), both excluding the NTR and 23andMe cohorts. After excluding these cohorts, the meta-analysis was redone for EA and ADHD symptoms. Since the NTR was present in the quantitative EAGLE summary statistics, which were combined with the Psychiatric Genetics Consortium (PGC) case–control ADHD summary statistics, we also re-applied the correction method to join case–control and quantitative summary statistics (Demontis et al. 2019).

**Fig. 1:**
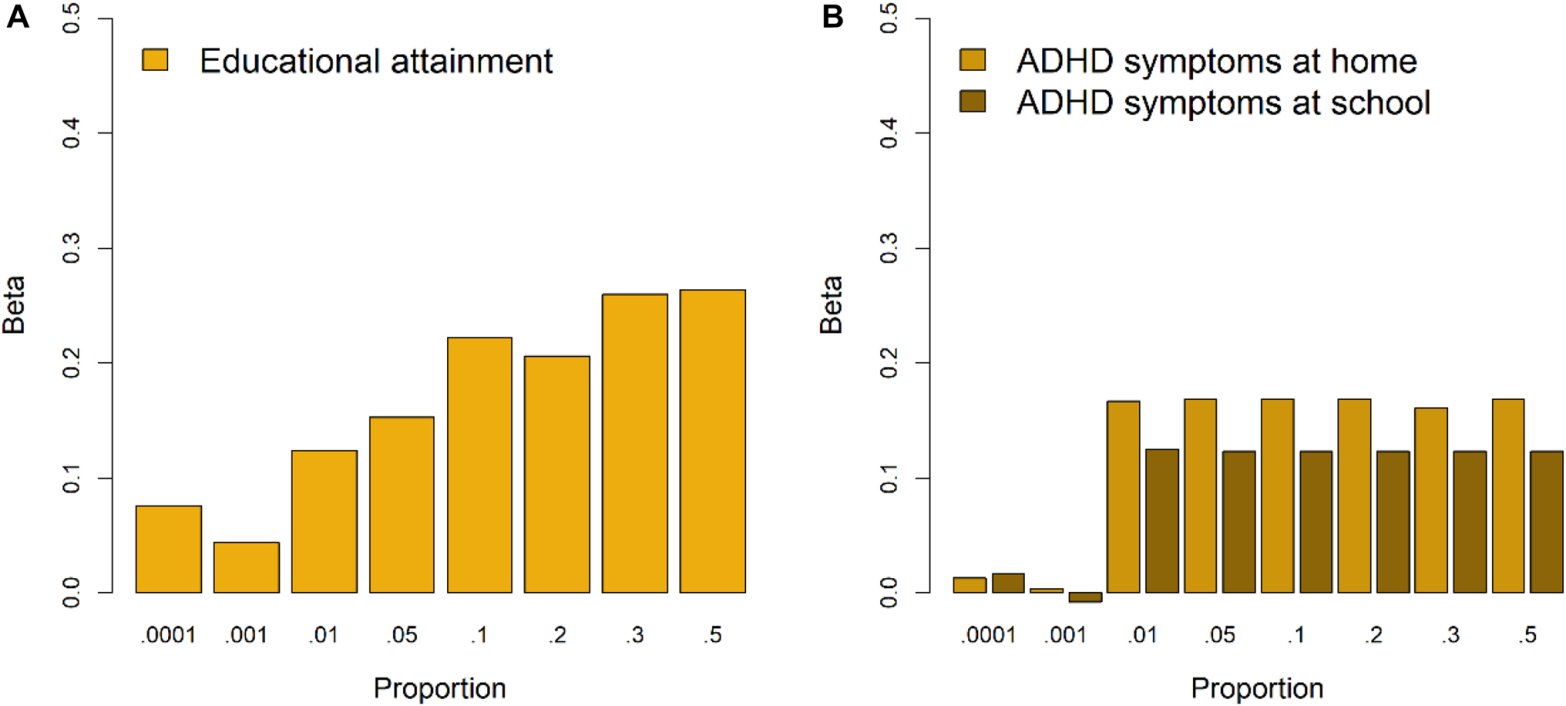
Effects of **a** the transmitted EA PGS on educational attainment in adults and of **b** the transmitted ADHD PGS on childhood ADHD symptoms at home and at school for different cut-offs of the proportion of causal markers

Based on these summary statistics sets, linkage disequilibrium (LD) weighted Beta's were calculated using the LDpred package with different cut-offs of the fraction of SNPs with a causal effect (Vilhjálmsson et al. 2015) with an LD pruning window of 250 KB (See Fig. 1). The reference population to calculate the LD patterns was a selection of the first 2500 2nd degree unrelated 1000G imputed individuals from the 5900 NTR individuals that were used for scoring. The detection of unrelated individuals was done with the King software (Manichaikul et al. 2010). The resulting LD corrected Beta's were used to calculate polygenic scores using the Plink 1.90 software, in the transmitted and non-transmitted alleles datasets.

### Statistical analyses

The current study included one offspring outcome in adulthood, i.e. EA, and two in childhood, i.e. academic achievement and ADHD symptoms. In adulthood, EA was regressed on the transmitted and non-transmitted EA PGS to test for replication of previous findings (Bates et al. 2018; Kong et al. 2018). In childhood, academic achievement, ADHD symptoms at home and ADHD symptoms at school were regressed on the transmitted and non-transmitted EA PGSs (model 1), on the transmitted and non-transmitted ADHD PGSs (model 2) and on the transmitted and non-transmitted EA plus ADHD PGSs (model 3). All outcome measures were residualized for the effects of sex, year of birth (only for EA), the interaction between sex and year of birth (only for EA), 10 principal components reflecting Dutch ancestry differences, and the genotyping platform. Within each analysis, the predictors and residualized outcome measures were standardized in the subset of individuals that had both PGS and phenotype data. A random intercept was added to correct for dependency of the observations due to family clustering. Generalized linear models were fitted in the statistical program SPSS Statistics for Windows 25.0 (IBM Corp. 2017) with maximum likelihood estimation. The type of model depended on the measurement level of the outcome: EA (ordinal logistic), academic achievement (linear) and ADHD symptoms (linear). To correct for multiple testing an alpha level of 0.01 was adopted.

### Power analysis

The sample included twin pairs and their siblings, which meant that observations were not independent. To facilitate the power analysis, we used the effective sample size, i.e. N_E_ = (N*M)/(1 + ICC*(M−1)) in which N = the number of families, M = the number of individuals in a family and ICC = the (average) phenotypic correlation within a family. We applied this separately for MZ and DZ (and siblings) families, given the expected differences in ICC. The power to detect a particular effect size (i.e. percentage of phenotypic variance explained) of the non-transmitted PGS was based on the non-central F-distribution. Power equals the percentage of significant tests of the regression coefficient given an alpha level of 0.01.

## Results

The EA and ADHD PGSs correlated moderately for both the transmitted (*r* = − 0.271) and non-transmitted (*r* =  − 0.234) PGSs, indicating that genes involved in EA and ADHD partly overlap. Note that this correlation cannot be interpreted as a genetic correlation in the traditional sense. The correlation between the transmitted and non-transmitted PGSs was low for both EA (*r* = 0.090) and ADHD (*r* = 0.033). PGSs and phenotype data were available for adult EA (N = 1931, level 0 = 0.5%, level 1 = 8.6%, level 2 = 32.0%, level 3 = 59.0%), and childhood academic achievement (N = 1120, Mean = 538.8, SD = 8.1, Range = 507–550), ADHD symptoms at home (N = 2518, Mean = 2.92, SD = 3.2, Range = 0–18) and ADHD symptoms at school (N = 1969, Mean = 6.09, SD = 7.7, Range = 0–43).

**Fig. 2:**
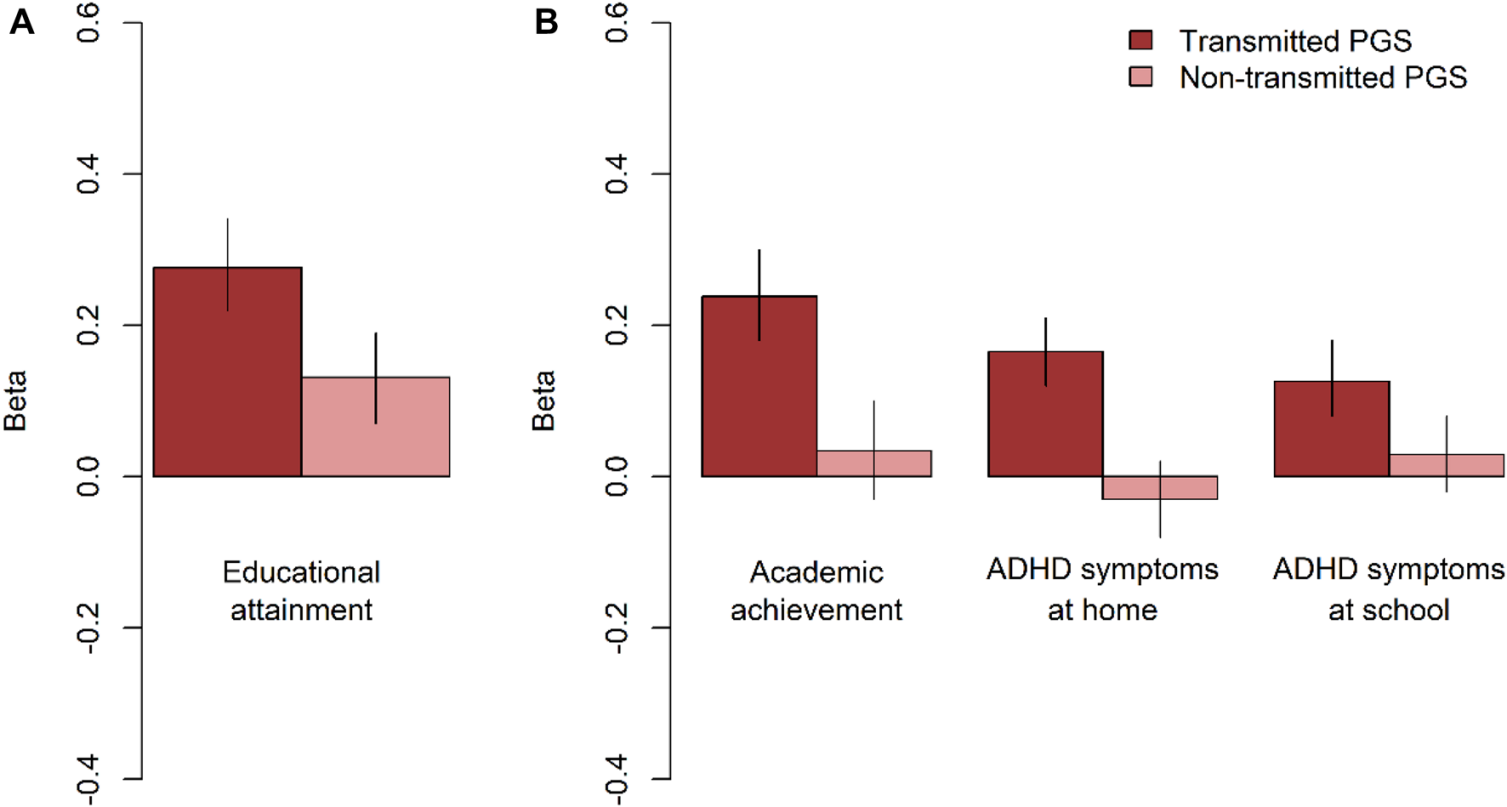
Effects (with 95% CI) of **a** the EA PGSs on educational attainment in adults and of **b** the EA PGSs on academic achievement in children and of the ADHD PGSs on childhood ADHD symptoms at home and at school

In adulthood, the transmitted (β = 0.28, p < 0.001) and non-transmitted (β = 0.13, p < 0.001) PGSs based on the EA meta-analysis (Lee et al. 2018) were significantly associated with EA (see Fig. 2a), replicating previous findings for genetic nurturing in EA (Bates et al. 2018; Kong et al. 2018). The magnitude of the estimated effect of the non-transmitted alleles for EA on adult EA was almost half of the effect of the transmitted alleles.

**Fig. 3:**
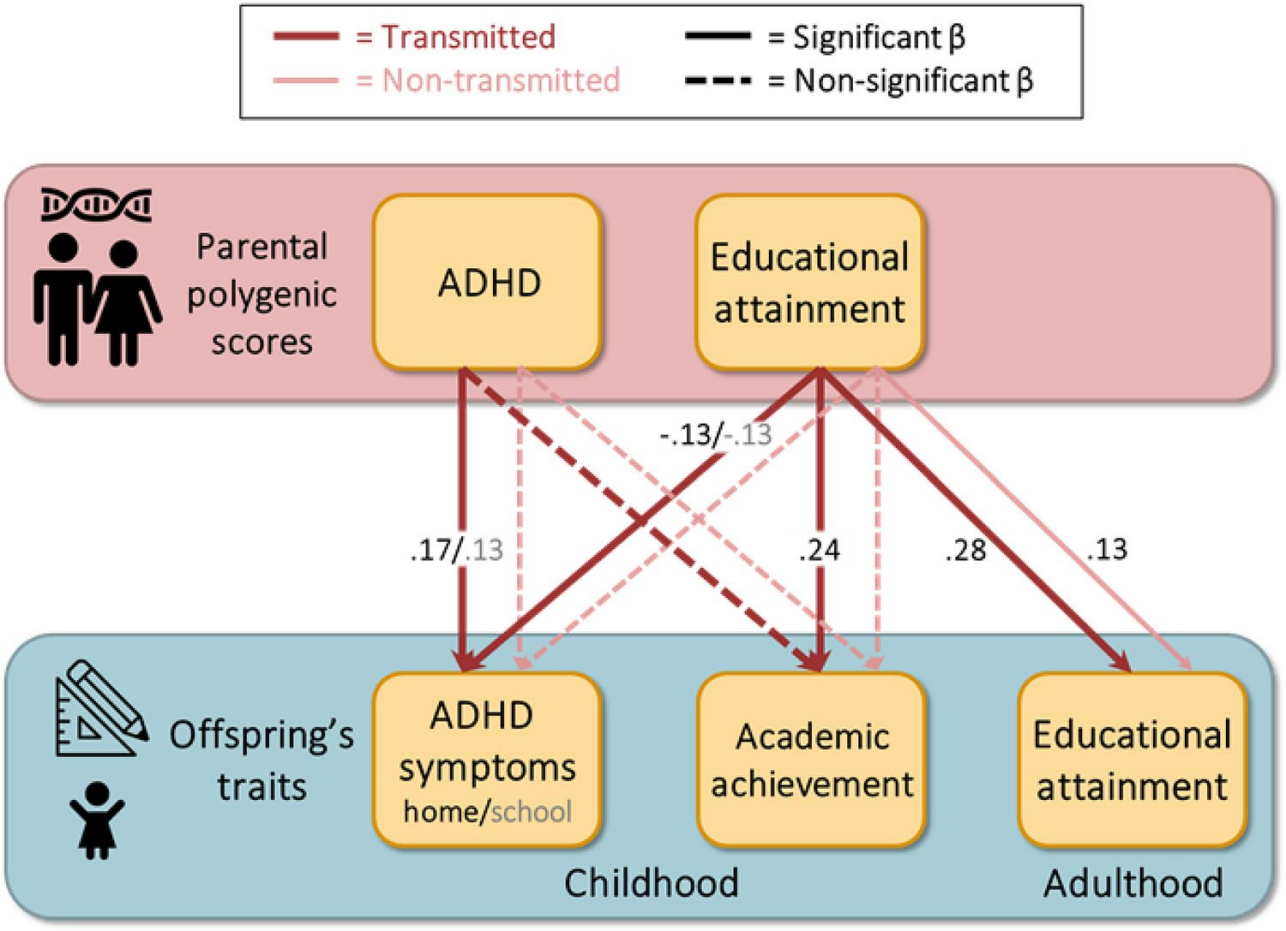
Schematic overview of the results. *Note* Dark lines represent the effect of transmitted polygenic scores and light lines represent the effect of non-transmitted polygenic scores. Solid lines represent a significant effect and dashed lines represent a non-significant effect. The depicted effects are the estimates for the regression coefficients as estimated for educational attainment and academic achievement in model 1 and for ADHD symptoms in model 2

In childhood, the transmitted EA PGS was significantly associated with academic achievement (β = 0.24, p < 0.001), ADHD symptoms at home (β =  − 0.13, p < 0.001) and ADHD symptoms at school (β =  − 0.13, p < 0.001) (Table 1—model 1). The non-transmitted EA PGS did not have a significant effect (see Fig. 2b). This suggests that there was no genetic nurturing on offspring’s academic achievement and ADHD symptoms elicited by parental EA. The transmitted ADHD PGS based on the ADHD meta-analysis (Demontis et al. 2019) was not associated with academic achievement (β =  − 0.08, p = 0.022), but had a significant influence on ADHD symptoms at home (β = 0.17, p < 0.001) and ADHD symptoms at school (β = 0.13, p < 0.001) (Table 1—model 2). The non-transmitted ADHD PGS was not associated with any of the outcomes, indicating that the environment that parents provided to their children based on the genes that play a role in their own ADHD symptoms did not affect their children’s development (see Fig. 2b). When taking the effects of both the EA and ADHD PGSs into account, the effect of the transmitted EA PGS (β = 0.23, p < 0.001) on academic attainment was similar, but the effect of the ADHD PGS (β =  − 0.02, p = 0.524) was attenuated. The effects of the transmitted EA PGS (β =  − 0.09, p < 0.001) and ADHD PGS (*β* = 0.14, p < 0.001) on ADHD symptoms at home were both diminished. The effects of the transmitted EA PGS (β = −0.10, p < 0.001) and ADHD PGS (*β* = 0.10, p < 0.001) on ADHD symptoms at school are also somewhat lower (Table 1—model 3). See Fig. 3 for a schematic representation of the results.

**Table 1:** The estimated effects (with 95% CI) of the transmitted (T) and non-transmitted (NT) polygenic scores for educational attainment (EA) and ADHD on offspring’s academic achievement, ADHD symptoms at home and ADHD symptoms at school

	Model 1 PGS_T_ EA + PGS_NT_ EA			Model 2 PGS_T_ ADHD + PGS_NT_ ADHD			Model 3 PGS_T_ EA + PGS_NT_ EA PGS_T_ ADHD + PGS_NT_ ADHD		
	Beta	R^2^ (%)	p	Beta	R^2^ (%)	p	Beta	R^2^ (%)	p
Academic achievement (N = 1120)									
PGS_T_ EA	0.238 (0.18; 0.30)	5.7	7 × 10^–15^				0.233 (0.17; 0.30)	5.4	2 × 10^–13^
PGS_NT_ EA	0.034 (− 0.03; 0.10)	0.1	0.284				0.033 (− 0.03; 0.10)	0.1	0.316
PGS_T_ ADHD				− 0.077 (− 0.14; − 0.01)	0.6	0.022	− 0.021 (− 0.09; 0.04)	0.0	0.524
PGS_NT_ ADHD				− 0.017 (− 0.08; 0.05)	0.0	0.610	− 0.006 (− 0.07; 0.06)	0.0	0.857
ADHD symptoms at home (N = 2518)									
PGS_T_ EA	− 0.125 (− 0.17; − 0.08)	1.6	4 × 10^–8^				− 0.088 (− 0.14; − 0.04)	0.8	2 × 10^–4^
PGS_NT_ EA	− 0.010 (− 0.06; 0.04)	0.0	0.669				− 0.017 (− 0.06; 0.03)	0.0	0.488
PGS_T_ ADHD				0.165 (0.12; 0.21)	2.7	2 × 10^–13^	0.141 (0.10; 0.19)	2.0	1 × 10^–9^
PGS_NT_ ADHD				− 0.030 (− 0.07; 0.02)	0.1	0.190	− 0.036 (− 0.08; 0.01)	0.1	0.127
ADHD symptoms at school (N = 1969)									
PGS_T_ EA	− 0.131 (− 0.18; − 0.08)	1.7	1 × 10^–7^				− 0.104 (− 0.16; − 0.05)	1.1	8 × 10^–5^
PGS_NT_ EA	− 0.012 (− 0.06; 0.04)	0.0	0.637				− 0.005 (− 0.06; 0.05)	0.0	0.848
PGS_T_ ADHD				0.126 (0.08; 0.17)	1.6	3 × 10^–7^	0.097 (0.05; 0.15)	0.9	2 × 10^–4^
PGS_NT_ ADHD				0.029 (− 0.02; 0.08)	0.1	0.243	0.024 (− 0.03; 0.08)	0.1	0.353

**Fig. 4:**
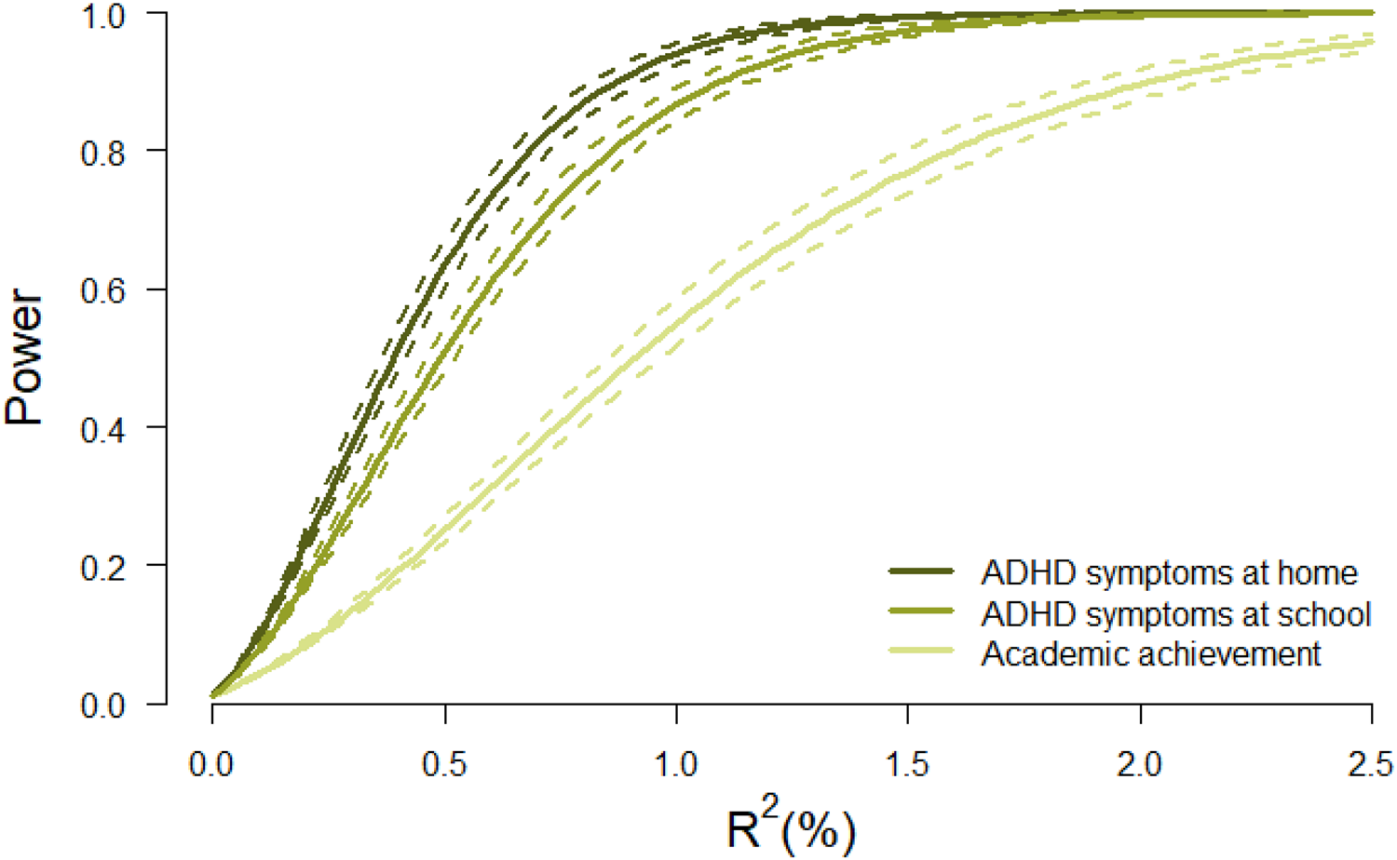
Power to detect the fixed effect of the non-transmitted PGS (expressed in R^2^) based on the calculated effective sample sizes for academic achievement (N_effective_ = 727), ADHD symptoms at home (N_effective_ = 1702) and ADHD symptoms at school (N_effective_ = 1352). *Note* Effective sample sizes are calculated with the formula (N*M)/(1 + ICC*(M−1)) in which *N * the number of families, *M * the number of individuals in a family and I*CC * the (average) phenotypic correlation within a family. Solid lines represent power with effective sample sizes calculated with the intraclass correlation (ICC) based on phenotypic correlations between family members for academic achievement (*r*_*MZ*_ = 0 .8; *r*_*DZ/SIB*_ = 0.4), ADHD symptoms at home (*r*_*MZ*_ = 0.8; *r*_*DZ/SIB*_ = 0.3) and ADHD symptoms at school (*r*_*MZ*_ = 0.8; *r*_*DZ/SIB*_ = 0.3). Dashed lines represent the power with effective sample sizes calculated with lower (*r−*0.1) and higher (*r* + 0.1) phenotypic correlations

To determine if we had enough power to detect an effect of genetic nurturing on our childhood outcomes, we carried out a series of power analyses. Figure 4 displays the power, based on the effective sample sizes (i.e. the number of independent cases corresponding to the number of clustered cases), to detect a fixed effect of the non-transmitted PGS on each of the outcomes. The analyses indicated that there was sufficient power (0.80) to detect an R^2^ explained by the non-transmitted PGS of 1.6%, 0.7% and 0.9% for, respectively, academic achievement, ADHD symptoms at home and ADHD symptoms at school (Fig. 3).

## Discussion

In the current study we showed that PGSs based on transmitted and non-transmitted alleles associated with EA impact adult’s lifetime EA, replicating findings of Bates et al. (2018) ad Kong et al. (2018). This demonstrates that adult EA is subject to genetic nurturing, i.e., the effects of parental alleles are mediated by parental behavior. In childhood, in contrast, we only found effects of transmitted alleles, despite having sufficient power to detect small effects (R^2^ = 0.7–1.6%) of non-transmitted alleles. The effect of genetic nurturing on ADHD was non-significant and close to zero (R^2^ ~ 0.1%). This supports our hypothesis concerning ADHD that genetic nurturing is not important. However, our hypothesis concerning academic achievement, namely that genetic nurturing would play a role in childhood academic achievement, was not supported. Regarding academic achievement, the twin literature shows a small, but significant, effect of common environment in the Netherlands (de Zeeuw et al. 2016), so we had expected to find a small, but significant, effect of genetic nurturing. However, Swagerman et al. (2017) found no evidence for environmental transmission in the parent – offspring resemblance in reading achievement. Possibly the common environment variance represents environmental influences shared by siblings that are independent of parent’s genes.

How can we reconcile the effect of genetic nurturing on education being absent in childhood (R^2^ = 0.1%), but present in adulthood (R^2^ = 1.7%)? First, we note that academic achievement scores at age 12 and the highest obtained degree in adulthood are different traits. We used PGSs for EA, i.e., the trait in adulthood. Hence, the effect of the transmitted alleles is expected to be somewhat higher for education in adulthood (7.6%) than childhood (5.7%). Nevertheless, this does not explain the difference in the effects of the non-transmitted alleles.

An increasing genetic nurturing effect is consistent with a slightly increasing influence of the common environment during secondary school (Rimfeld et al. 2018), and a substantial influence of the common environment in adults’ EA (Branigan et al. 2017). We speculate that the common environmental effect (and genetic nurturing effect) increases in secondary school due to educational tracking. Tracking in the Netherlands takes place from age 12 onwards, partly based on the academic achievement test that we analyzed here. Parents’ EA was still associated with test scores after accounting for genetic influences (de Zeeuw et al. 2019). Moreover, compared to children of low-educated parents, children of high EA parents are more likely to enroll in and complete a higher educational track than expected based solely on their academic achievement test result (van Spijker et al. 2017).

The role of parental EA in their offspring EA seems to exceed what can be expected on the basis of the inherited genes. This is in line with findings from the UK, in which the common environment substantially influences educational choice (Rimfeld et al. 2016; Smith-Woolley et al. 2018). Next, due to tracking in the Netherlands, the children’s secondary school environment varies more that their primary school environment. This source of greater environmental variance may increase the contribution of the common environment to achievement differences. In addition, again due to tracking, children’s school level is approximately matched to their realized genetic potential, thereby inducing a gene–environment correlation. In the classical twin design with uncorrelated genetic and common environmental factors, this correlation will inflate the estimate of common environment variance.

Given the lack of common environment in the ADHD literature (Faraone and Larsson 2019) and the large contribution of dominant genetic effects (Rietveld et al. 2003; Nikolas and Burt 2010), we expected no, and indeed found no, genetic nurturing in childhood ADHD. Our confidence in this null-result for ADHD is strengthened by the fact we had sufficient power (0.80) to detect relatively small effects (at home 0.7% and at school 0.9%), but found no effect of genetic nurturing in either the home or the school environment. Apparently, the aspects of the home environment that are associated with parents genetic ADHD liability do not impact children’s cognitive and behavioral development.

We set out to investigate transmission of EA to outcomes both within and between domains. In childhood, only effects of transmitted PGSs were significant, so all associations were attributable to genetic transmission, and not genetic nurturing. Children’s ADHD symptoms were associated with both PGSs based on transmitted alleles associated with ADHD and EA. This is consistent with the genetic correlation between academic achievement and ADHD (e.g. Liu et al. 2019). Academic achievement was only influenced by parents’ EA associated alleles. In light of the current results, earlier reported associations between household chaos and academic achievement (Johnson et al. 2009; Hanscombe et al. 2011) would seem to have a genetic rather than an environmental source. However, this is not in line with a British twin study which suggested that the association between chaotic homes and poor academic achievement was due to the combination of shared genetic and environmental effects (Hanscombe et al. 2011). These seemingly conflicting conclusions may be due to population differences: the Dutch educational system is egalitarian, resulting in reduced effects of the common environment (Kovas et al. 2013; de Zeeuw et al. 2016). The educational system in the Netherlands is similar to the one in the UK as both countries have a national curriculum and schools comply to governmental standards. However, in the UK there are state-funded schools and private schools (~ 7%) (Britisch Educational Suppliers Association 2017), which charge tuition and have a selective admittance policy. In the Netherlands, on the other hand, the few private schools (~ 1%) are not allowed to select students (Ministerie 2018). In addition, the Gini index, which measures income inequality within a country, is higher in the UK (33.2) compared to the Netherlands (28.2) (World Bank 2015).

A significant effect of the non-transmitted PGS demonstrates that parental behavior related to EA matters. However, the absence of an effect of the non-transmitted PGS does not necessarily mean that these associated parenting behaviors do not matter. It is possible that the link between the education related parenting behaviors and childhood academic achievement is attributable to the transmission of genetic effects. The effects of parenting behaviors not associated with EA can, however, still influence the environment and subsequently a child’s behavior, or in other words, genetic nurturing. These parental behaviors could be shaped by other parental traits with a low genetic correlation with EA, such as personality traits, well-being, and health-related traits (Bulik-Sullivan et al. 2015). Adding non-transmitted PGSs related to these other parental characteristics may help characterize a child’s environment, and to identify the parental behaviors that have an impact on offspring development.

It was recently demonstrated that passive gene–environment correlations inflate the predictive power of PGSs for cognitive traits but not for ADHD. Selzam et al. (2019) leveraged the fact that within-family analyses account for passive gene–environment correlations. In contrasting within- and between-family analyses, it was shown that PGS association estimates for cognitive traits were greater between families than within families. In contrast, within- and between-family estimates were similar for the association with ADHD. Passive gene–environment correlations are picked up in our design by genetic nurturing. Therefore, their conclusion is compatible with our finding that genetic nurturing contributes to EA, but not to ADHD.

The use of PGSs based on GWAS results in the genetic nurturing design means that GWAS limitations carry over to the current study, including the limitation that residual population stratification may affect the summary statistics, variation in phenotype definitions in discovery GWAS samples, and differences in populations (De La Vega and Bustamante 2018). In addition, what must be noted is that for EA the number of females is double that of the number of males. If the effect of the home environment is larger in females, which might be the case in older generations (Branigan et al. 2017), this could have overestimated the effect of genetic nurturing. Nonetheless, the genetic nurturing design has unique strengths. It can demonstrate influences of the family environment without relying on self-reports. Studies on, for example, the impact of growing up in a chaotic household typically rely on parents’ own judgement of their home situation or of their own ADHD symptoms. Here we used ADHD and EA related genetic variation as a tool to demonstrate causal environmental effects un-confounded by genetic effects. This design requires the presence of measured genotypes and phenotypes in the offspring, and measured genotypes in the parents. However, adding parental phenotypes would create possibilities to further disentangle effects of direct genetic liabilities, causal environments and forms of gene–environment correlation. The addition of parental phenotypes facilitates the identification of the processes underlying genetic nurturing. For example, one may address the question whether the effects of genetic nurturing are due to parental cognitive stimulation or household chaos (Wertz 2019).

To summarize, we found a genetic effect mediated by the environment (‘genetic nurturing’) on EA, in addition to the direct genetic effect, in adulthood. For academic achievement and ADHD in childhood, we only found evidence for direct genetic effects. We can thus conclude that reported associations between home characteristics related to parental EA and ADHD and child outcomes seem to be mainly a marker of genetic effects shared by parents and children.

## References

[CR1] Achenbach T, Rescorla L (2001). Manual for the ASEBA school-age forms & profiles.

[CR2] Achenbach TM, Ivanova MY, Rescorla LA (2017). Empirically based assessment and taxonomy of psychopathology for ages 1½–90+ years: developmental, multi-informant, and multicultural findings. Compr Psychiatry.

[CR3] Bates TC, Maher BS, Medland SE (2018). The nature of nurture: using a virtual-parent design to test parenting effects on children’s educational attainment in genotyped families. Twin Res Hum Genet.

[CR4] Belsky D, Domingue B, Wedow R (2018). Genetic analysis of social mobility in five longitudinal studies. Proc Natl Acad Sci.

[CR5] Boomsma DI, Saviouk V, Hottenga JJ (2010). Genetic epidemiology of attention deficit hyperactivity disorder (ADHD index) in adults. PLoS ONE.

[CR6] Boomsma DI, Wijmenga C, Slagboom EP (2014). The genome of the Netherlands: design, and project goals. Eur J Hum Genet.

[CR7] Branigan AR, Mccallum KJ, Freese J (2017). Variation in the heritability of educational attainment : An international meta-analysis. Soc Forces.

[CR8] Britisch Educational Suppliers Association (2017) Key UK education statistics

[CR9] Bulik-Sullivan B, Finucane HK, Anttila V (2015). An atlas of genetic correlations across human diseases and traits. Nat Genet.

[CR10] Chevalier A, Harmon C, O’Sullivan V, Walker I (2013). The impact of parental income and education on the schooling of their children. IZA J Labor Econ.

[CR11] Cito (2002). Eindtoets basisonderwijs.

[CR12] Coldwell J, Pike A, Dunn J (2006). Household chaos - links with parenting and child behaviour. J Child Psychol Psychiatry Allied Discip.

[CR14] D’Onofrio BM, Turkheimer EN, Eaves LJ (2003). The role of the children of twins design in elucidating causal relations between parent characteristics and child outcomes. J Child Psychol Psychiatry Allied Discip.

[CR15] Das S, Forer L, Schönherr S (2016). Next-generation genotype imputation service and methods. Nat Genet.

[CR16] De La Vega FM, Bustamante CD (2018). Polygenic risk scores: a biased prediction?. Genome Med.

[CR17] de Zeeuw EL, van Beijsterveldt CEM, Glasner TJ (2014). Polygenic scores associated with educational attainment in adults predict educational achievement and ADHD symptoms in children. Am J Med Genet Part B.

[CR18] de Zeeuw EL, van Beijsterveldt CEM, Glasner TJ (2016). Arithmetic, reading and writing performance has a strong genetic component: a study in primary school children. Learn Individ Differ.

[CR19] de Zeeuw E, Kan K-J, van Beijsterveldt C (2019). The moderating role of SES on genetic differences in educational achievement in the Netherlands. NPJ Sci Learn.

[CR20] Demontis D, Walters RK, Martin J (2019). Discovery of the first genome-wide significant risk loci for attention deficit/hyperactivity disorder. Nat Genet.

[CR21] Faraone SV, Larsson H (2019). Genetics of attention deficit hyperactivity disorder. Mol Psychiatry.

[CR22] Galinsky KJ, Bhatia G, Loh PR (2016). Fast principal-component analysis reveals convergent evolution of ADH1B in Europe and East Asia. Am J Hum Genet.

[CR23] Hanscombe KB, Haworth CMA, Davis OSP (2011). Chaotic homes and school achievement: a twin study. J Child Psychol Psychiatry Allied Discip.

[CR24] Hart S, Little C, van Bergen E (2019) Nurture might be nature: cautionary tales and proposed solutions. preprint 10.1038/s41539-020-00079-z PMC7794571 33420086

[CR13] IBM Corp. (2017). IBM SPSS statistics for windows.

[CR25] Johnson A, Martin A, Brooks-Gunn J, Petrill S (2009). Order in the House! Associations among household chaos, the home literacy environment, maternal reading ability, and children’s early reading. Merrill Palmer Q.

[CR26] Kong A, Thorleifsson G, Frigge ML (2018). The nature of nurture: effects of parental genotypes. Science.

[CR27] Kovas Y, Voronin I, Kaydalov A (2013). Literacy and numeracy are more heritable than intelligence in primary school. Psychol Sci.

[CR28] Lee JJ, Wedow R, Okbay A (2018). Gene discovery and polygenic prediction from a genome-wide association study of educational attainment in 1.1 million individuals. Nat Genet.

[CR29] Ligthart L, van Beijsterveldt CEM, Kevenaar ST (2019). The Netherlands Twin Register: longitudinal research based on twin and twin-family designs. Twin Res Hum Genet.

[CR30] Liu CY, Li Y, Viding E (2019). The developmental course of inattention symptoms predicts academic achievement due to shared genetic aetiology: a longitudinal twin study. Eur Child Adolesc Psychiatry.

[CR31] Lundborg P, Nordin M, Rooth D-O (2018). The intergenerational transmission of human capital: exploring the role of skills and health using data on adoptees and twins. J Popul Econ.

[CR32] Manichaikul A, Mychaleckyj JC, Rich SS (2010). Robust relationship inference in genome-wide association studies. Bioinformatics.

[CR33] McAdams TA, Neiderhiser JM, Rijsdijk FV (2014). Accounting for genetic and environmental confounds in associations between parent and child characteristics: a systematic review of children-of-twins studies. Psychol Bull.

[CR34] McGue M, Rustichini A, Iacono WG (2017). Cognitive, noncognitive, and family background contributions to college attainment: a behavioral genetic perspective. J Pers.

[CR35] Mol SE, Bus AG (2011). To read or not to read: a meta-analysis of print exposure from infancy to early adulthood. Psychol Bull.

[CR36] Nikolas MA, Burt SA (2010). Genetic and environmental influences on ADHD symptom dimensions of inattention and hyperactivity: a meta-analysis. J Abnorm Psychol.

[CR37] Plomin R, Bergeman CS (1991). The nature of nurture: genetic influence on “environmental” measures. Behav Brain Sci.

[CR38] Polderman TJC, Boomsma DI, Bartels M (2010). A systematic review of prospective studies on attention problems and academic achievement: review. Acta Psychiatr Scand.

[CR39] Purcell S (2002). Variance components models for gene–environment interaction in twin analysis. Twin Res.

[CR40] Rietveld MJH, Posthuma D, Dolan CV, Boomsma DI (2003). ADHD: sibling interaction or dominance: an evaluation of statistical power. Behav Genet.

[CR41] Rimfeld K, Ayorech Z, Dale PS (2016). Genetics affects choice of academic subjects as well as achievement. Sci Rep.

[CR42] Rimfeld K, Malanchini M, Krapohl E (2018). The stability of educational achievement across school years is largely explained by genetic factors. NPJ Sci Learn.

[CR58] Selzam S, Ritchie SJ, Pingault J-B, Reynolds CA, O’Reilly PF, Plomin R (2019). Comparing Within- and Between-Family Polygenic Score Prediction. Am J Hum Genet.

[CR43] Sikora J, Evans MDR, Kelley J (2019). Scholarly culture: how books in adolescence enhance adult literacy, numeracy and technology skills in 31 societies. Soc Sci Res.

[CR44] Silberg J, Maes H, Eaves L (2012). Unraveling the effect of genes and environment in the transmission of parental antisocial behavior to children’s conduct disturbance, depression, and hyperactivity. J Child Psychol Psychiatry.

[CR45] Smith-Woolley E, Ayorech Z, Dale PS (2018). The genetics of university success. Sci Rep.

[CR46] Stergiakouli E, Martin J, Hamshere ML (2017). Association between polygenic risk scores for attention-deficit hyperactivity disorder and educational and cognitive outcomes in the general population. Int J Epidemiol.

[CR57] Swagerman SC, van Bergen E, Dolan C, de Geus EJC, Koenis MMG, Hulshoff Pol HE, Boomsma DI (2017). Genetic transmission of reading ability. Brain Lang.

[CR47] van Ministerie OCW (2018). Onderwijs in cijfers.

[CR49] van Bergen E, van Zuijen T, Bishop D, de Jong PF (2017). Why are home literacy environment and children’s reading skills associated? What parental skills reveal. Read Res Q.

[CR50] van Boxtel H, Engelen R, de Wijs A (2010). Wetenschappelijke verantwoording van de Eindtoets Basisonderwijs.

[CR51] van Spijker F, van der Houwen K, van Gaalen R (2017). Invloed ouderlijk in het voortgezet onderwijs. ESB.

[CR52] Vilhjálmsson BJ, Yang J, Finucane HK (2015). Modeling linkage disequilibrium increases accuracy of polygenic risk scores. Am J Hum Genet.

[CR53] Vinkhuyzen AAE, Van Der Sluis S, De Geus EJC (2010). Genetic influences on “environmental” factors. Genes, Brain Behav.

[CR54] Wertz J (2019). Using DNA from mothers and children to study parental investment in children’s educational attainment. bioRxiv.

[CR56] World Bank (2015) GINI index

